# Reprogramming of spermatogonial stem cells into pluripotent stem cells in the spheroidal state

**DOI:** 10.1080/19768354.2019.1672578

**Published:** 2019-10-08

**Authors:** Yukyeong Lee, Minseong Lee, Seung-Won Lee, Na Yong Choi, Seokbeom Ham, Hye Jeong Lee, Kisung Ko, Kinarm Ko

**Affiliations:** a Department of Stem Cell Biology, Konkuk University School of Medicine, Seoul, Korea; b Center for Stem Cell Research, Institute of Advanced Biomedical Science, Konkuk University, Seoul, Korea; c Viral Disease Division, Animal and Plant Quarantine Agency, Gimcheon, Korea; d Department of Medicine, College of Medicine, Chung-Ang University, Seoul, Korea; e Research Institute of Medical Science, Konkuk University, Seoul, Korea

**Keywords:** Spermatogonial stem cells, pluripotent stem cells, 3D culture

## Abstract

Spermatogonial stem cells (SSCs) are unipotent adult stem cells, capable of differentiating into sperm cells. SSCs can be cultured *in vitro* for a long time. SSCs expressing Oct4, a pluripotency marker, and are the only adult cells which pluripotency can be induced under defined culture conditions. However, because 2D culture imposes limitations in cell junction formation, cell shape, metabolism, response to stimuli, and cell interface with medium, mechanistic studies on reprogramming of SSCs using feeder cells still have many challenges. Recent studies have shown that a culture system using a bio-matrix can be used in long-term feeder-free SSCs culture and for induction of pluripotency in SSCs. However, the bio-matrix cannot be the optimal micro-environment in mechanistic studies because it creates a physical barrier to growth factors and other signaling molecules. To overcome this effect of the matrix, we reprogrammed SSCs into pluripotent ESC-like cells, so-called germline-derived pluripotent stem cells (gPSCs) by using a 3D scaffold, in which cells are less responsive to external stimuli than in 2D cultures. Thus, we confirm the possibility of SSC reprogramming in the spheroidal state and suggest the utility of 3D scaffolds as a tool for studying the mechanism of SSC reprogramming into gPSCs without a bio-matrix.

## Introduction

Stem cell differentiation and reprogramming techniques are rapidly developing. Recently, neural stem cells and hepatocytes have been produced by direct conversion from fibroblasts without inducing pluripotency (Sekiya and Suzuki 2011; Han et al. 2012). In addition, experiments are underway to understand signaling between different types of cells using co-culture in 3D scaffolds (Kook et al. 2017). These biomimetic 3D scaffolds reproduce the niches of various cells, enabling the culture and analysis of various types of cells that cannot be done in the 2D culture (van Neerven et al. 2014). 2D culture substrates not only fall short of reproducing the complex and dynamic environments of the body but also are likely to misrepresent findings to some degree because they force the cells to adjust to an artificial flat and rigid surface. For these reasons, 3D scaffolds were introduced to overcome 2D culture limitations such as cell junction formation, cell shape, metabolism, response to stimuli, and cell interface with medium (Lee et al. 2008).

Spermatogonial stem cells (SSCs) are adult stem cells capable of differentiating into sperm cells. SSCs are an extremely rare population, only 0.03% of all germ cells in the testis (Tegelenbosch and de Rooij 1993). Unlike other adult stem cells, SSCs expressing the pluripotency marker octamer-binding transcription factor4 (Oct4) and can be reprogrammed under defined culture conditions (Kanatsu-Shinohara et al. 2004; Kehler et al. 2004; Dann et al. 2008; Ko et al. 2009). A recent study found that pluripotency can be induced in SSCs in bio-matrix-coated dishes even in the absence of feeder cells (Lee et al. 2018). This proves that pluripotency can be induced in homogeneous SSC populations without other cells. Nonetheless, a bio-matrix is made from cell-derived extracts and contains a variety of cell-based materials. Because these materials can affect SSC reprogramming, their effect should be minimized. Therefore, we used a 3D scaffold in this study to induce pluripotency in SSC in the absence of a bio-matrix and generated so-called germline-derived pluripotent stem cells (gPSCs). We demonstrated that pluripotency can be induced in SSCs in a spheroidal state in 3D scaffolds. This method avoids the effect of exogenous cell extracts on SSC reprogramming. Furthermore, this study suggests a platform for studying the mechanism of reprogramming into gPSCs using 3D scaffolds.

## Materials and methods

### Culture medium for SSC expansion

Establishment of SSCs from Oct4-GFP/LacZ and Oct4-GFP transgenic mice (C57BL/6 background) was described previously (Ko et al. 2009; Kanatsu-Shinohara et al. 2011; Han et al. 2012). SSC medium for expansion was composed of StemPro-34 SFM (Gibco, Carlsbad, CA, USA) with the following supplements: StemPro (Gibco), 1× N2 (Gibco), 6 mg/ml d-(+)-glucose (Gibco), 30 mg/ml pyruvic acid (Gibco), 1 μl/ml DL-lactic acid (Sigma-Aldrich, Saint Louis, MO, USA), 5 mg/ml bovine serum albumin (BSA; Gibco), 1% fetal bovine serum (FBS; Gibco), 2 mM L-glutamine (Gibco), 50 μM β-mercaptoethanol (Gibco), 1×penicillin/streptomycin (Welgene, Gyeongsan, Korea), 1× minimal essential medium (MEM) with non-essential amino acids (Gibco), 1× MEM vitamins (Welgene), 30 ng/ml β-estradiol (Sigma), 60 ng/ml progesterone (Sigma), 20 ng/ml human EGF (Peprotech, Rocky Hill, NJ, USA), 20 ng/ml human bFGF (Peprotech), 20 ng/ml human GDNF (Peprotech), and 10^3^ U/ml murine leukemia inhibitory factor (Prospec, Rehovot, Israel).

### 3D Scaffold

3D Stemfit^®^ culture dishes (3D scaffold) were purchased from manufacturer (MicroFIT, Seongnam, Korea). The internal structure of the wells of 3D scaffold was illustrated in Supplementary Figure 1. The scaffold is composed of 853 wells with a diameter of 400 μm and a depth of 450 μm per well. For reprogramming experiment, 3D scaffold was attached to 6-well plates.

### Induction of pluripotency in the spheroidal state

Bubbles in the 3D scaffold were removed with 1 ml of 70% ethyl alcohol (EtOH), and their absence was confirmed under a microscope. The scaffold was then washed three times with SSC medium to remove EtOH. SSCs (1 × 10^6^) were seeded in the 3D scaffold. After 30 min, medium was containing spheroid-defective cells was removed and fresh medium was added. The medium was exchanged every 2 days.

### PCR

Total RNA was isolated by using an RNeasy Mini Kit (Qiagen, Hilden, Germany) according to the manufacturer’s instructions. Total RNA (500 ng) was reverse-transcribed by using an OmniscriptRT Kit (Qiagen) in a total volume of 20 μl. PCR analysis was performed with gene-specific primers and Takara Ex Taq DNA polymerase (Takara Bio inc., Kusatsu, Shiga, Japan) according to the manufacturer’s instructions. The PCR conditions were as follows: 32 cycles at 94°C for 30 s, 50–65°C for 30 s, and 72°C for 30 s. The RT–PCR products were analyzed by electrophoresis in 1% agarose gels. Primer sequences are listed in Supplementary Table 1.

### Genomic DNA isolation and bisulfite treatment

Genomic DNA was isolated using a G-spin total DNA extraction kit (Intron, Liberty Lake, WA, USA). Genomic DNA (1 μg) was bisulfite converted using an Epi-tect Bisulfite Kit (Qiagen) according to the manufacturer’s instructions.

### Bisulfite sequencing analysis

Bisulfite-converted DNA was used for PCR with Hot-StarTaq (Qiagen). Primers sequences are listed in Supplementary Table 2. PCR products were cloned using the PCR Cloning kit (Qiagen). Quantification Tool for Methylation Analysis (QUMA) was used for methylation analysis.

### Immunocytochemistry

Cells were fixed in 4% paraformaldehyde (Sigma-Aldrich, US) for 15 min at room temperature (RT), washed three times with dulbecco’s phosphate buffered saline (DPBS; Hyclone, Logan, UT, USA), and then incubated in DPBS containing 0.5% Triton X-100 (Sigma-Aldrich), 1% BSA fraction V (Sigma-Aldrich), and 10% FBS for 10 min at RT. The cells were briefly rinsed with DPBS and incubated with primary antibodies overnight at 4°C or for 1 h at RT. The following primary antibodies were used: mouse monoclonal anti-SSEA1 (1:200, DSHB, Iowa, IA, USA), mouse monoclonal anti-microtubule-associated protein-2 (anti-Map2; 1:300, R&D Systems Inc., Minneapolis, MN, USA), mouse monoclonal anti-spinal muscular atrophy (anti-SMA; 1:200, R&D Systems Inc.) and mouse monoclonal anti-alpha-fetoprotein (anti-AFP; 1:200, R&D Systems Inc.). The cells were then washed with 0.5% BSA in PBS and incubated with secondary antibodies (1:1000, R&D System Inc.) for 1 h at RT. Nuclei were detected by 4'-6-Diamidino-2-phenylindole (DAPI) antibody (1:1000, Sigma-Aldrich) staining.

### *In vitro* differentiation of scaffold-gPSCs (SF-gPSCs)

To differentiate SF-gPSCs into three germ layers, previously described protocols (Brustle et al. 1999; Igelmund et al. 1999) were applied to embryoid bodies derived from gPSCs. Embryoid bodies were attached to gelatin coated plates and cultured in MEF medium until beating cells formed. MEF medium was composed of low-glucose DMEM (Welgene) with the following supplements: 10% FBS, 50 μM β-mercaptoethanol, 1×penicillin/streptomycin, and 1×(MEM) non-essential amino acids.

### Teratoma formation for *in vivo* differentiation of SF-gPSCs

SF-gPSCs were transplanted into immunodeficient mice and all mice were sacrificed at 10 weeks of transplantation. The teratomas were dissected, fixed with Bouin’s solution and embedded in paraffin. Paraffin sections were stained with hematoxylin and eosin.

## Results

### Induction of pluripotency in the spheroidal state

**Figure 1.**
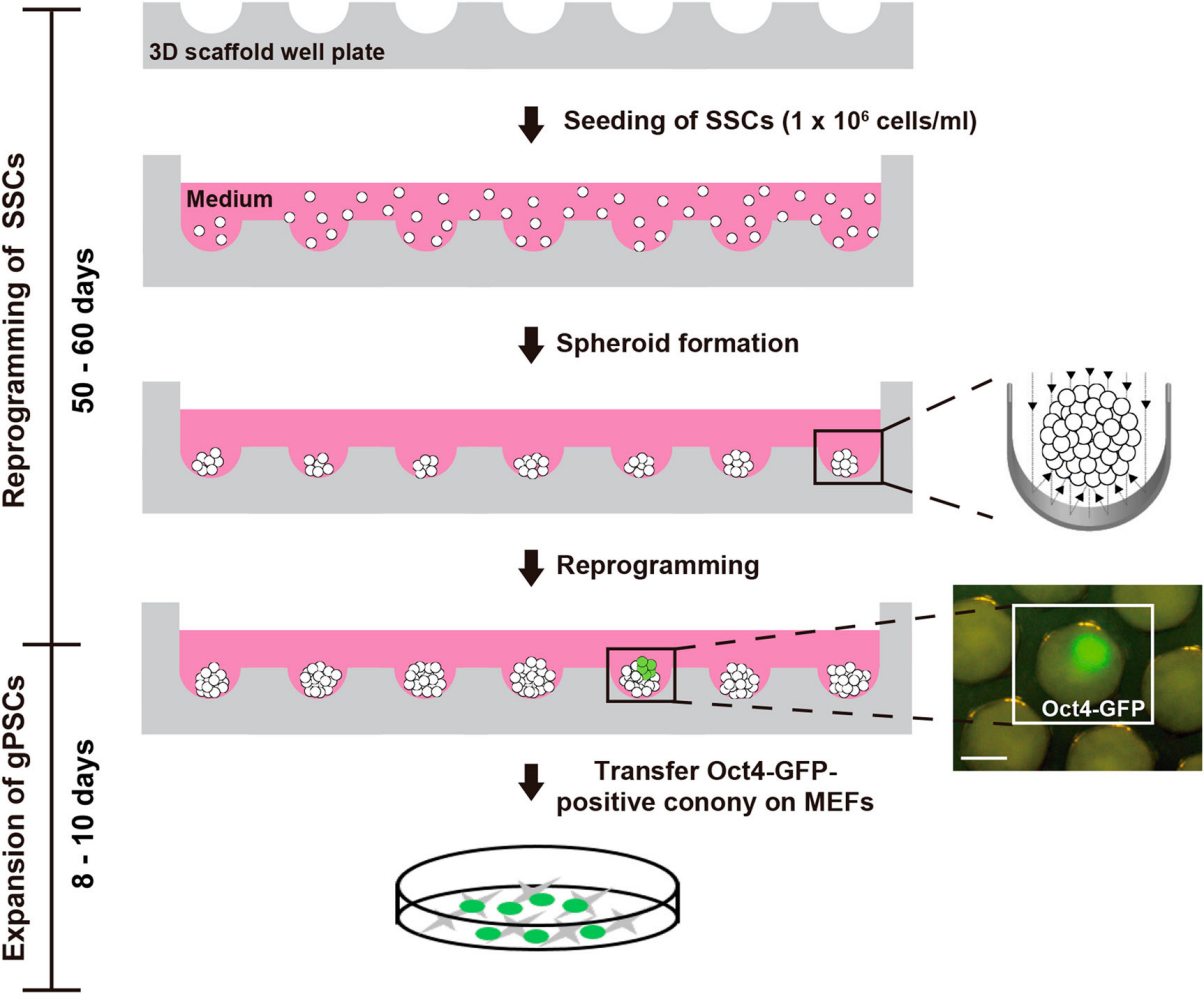
Schematic diagram of SF-gPSCs generation from SSCs using a 3D scaffold. Scale bar: 200 μm.

**Figure 2.**
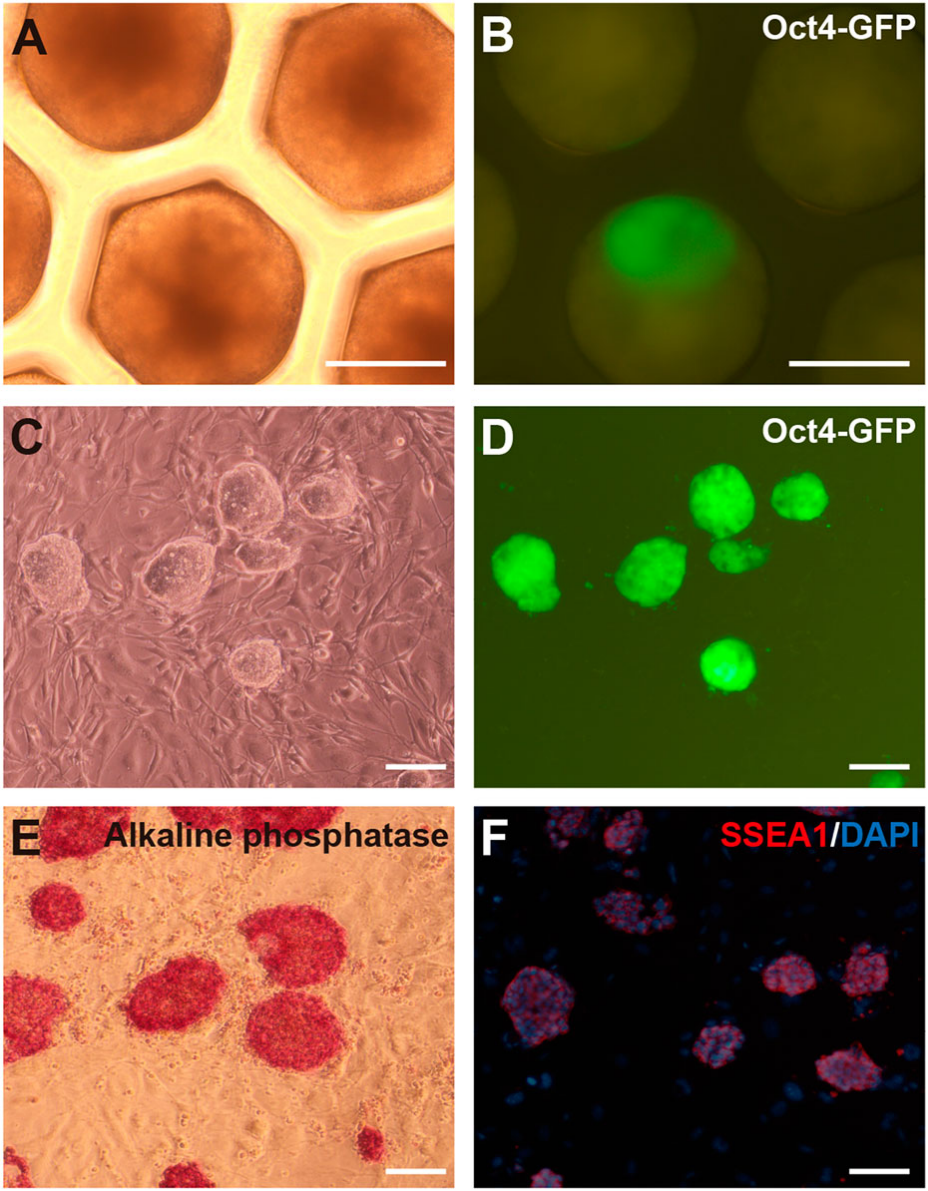
Induction of SF-gPSCs. (A, B) Representative (A) phase contrast and (B) GFP-positive images of the conversion of SSCs into gPSCs in a 3D scaffold. (C, D) Representative (C) phase contrast and (D) GFP-positive images of SF-gPSCs from Oct4-GFP-expressing colonies. (E) Immunofluorescence staining of alkaline phosphastase in SF-gPSCs. (F) SSEA1 staining in SF-gPSCs. Scale bars: 200 μm (A–G).

In the 3D scaffold, SSCs formed spheroids (Figure 1A). Oct4-GFP-positive colony in 1 well or 2 wells out of 853 wells was observed among SSC spheroids after 50–60 days (Figures 1 and 2(B)). They expressed high levels of Oct4-GFP and showed the embryonic stem cells (ESCs)-like morphology cultured on feeder cells (Figure 2(C,D)). We repeated the experiment three times with 1 × 10^6^ cells per scaffold. Two gPSC lines in 3D scaffold (SF-gPSCs) were established from the observed Oct4-positive colonies in scaffold. SF-gPSCs stained positive for alkaline phosphatase and SSEA-1 (Figure 2(E,F)).

### Gene expression profile in SF-gPSCs is similar to that in ESCs and gPSCs

**Figure 3.**
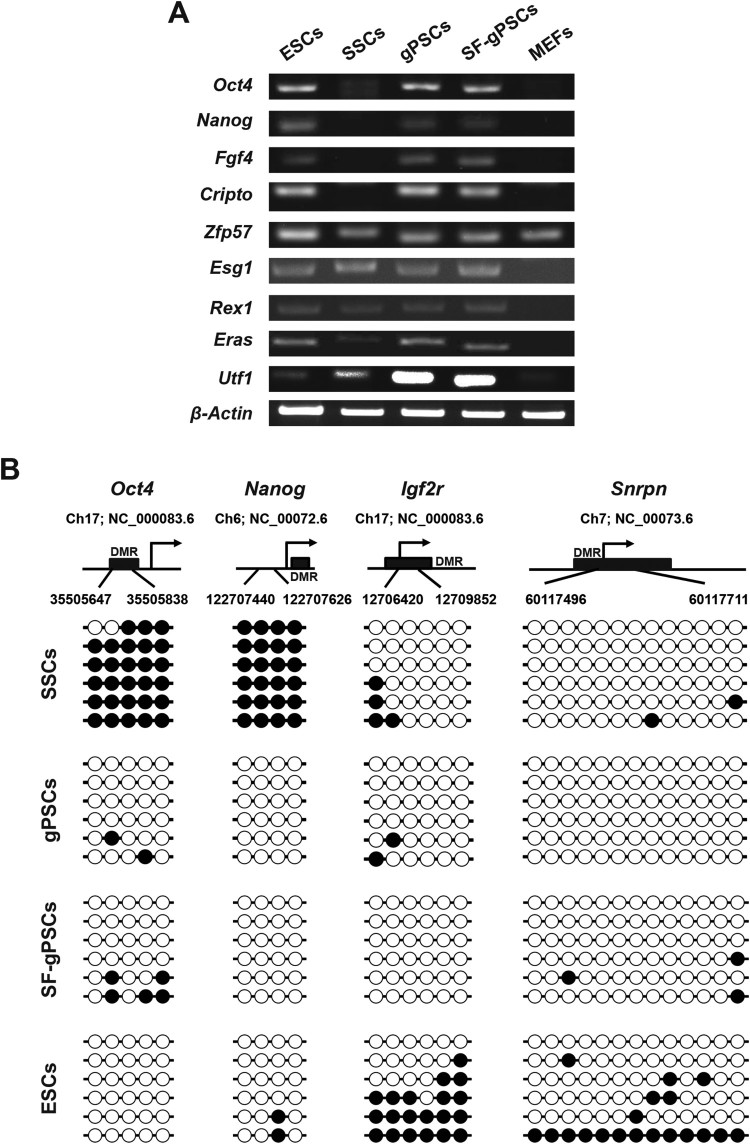
RT-PCR analysis of pluripotency marker gene expression and DNA methylation analysis. (A) Expression of pluripotency marker genes was analyzed by RT-PCR in ESCs, SSCs, gPSCs, SF-gPSCs, and MEFs. (B) DNA methylation patterns of *Oct4*, *Nanog* and the maternally methylated genes *Snrpn* and *Igf2r* in ESCs, SSCs, gPSCs, and SF-gPSCs. Each line represents a single clone. Black and white circles represent methylated and unmethylated CpGs, respectively.

RT–PCR analysis revealed that the expression of the pluripotency marker genes *Oct4*, *Homeobox transcription factor nanog* (*Nanog)*, *Fibroblast growth factor 4* (*Fgf4)*, *Teratocarcinoma-derived growth factor 1* (*Cripto)* *, Zinc finger protein 57* (*Zfp57)*, *Enhancer of split groucho-like protein 1* (*Esg1)*, *Reduced expression protein1* (*Rex1)*, *ES cell expressed ras* (*Eras)*, and *Undifferentiated embryonic cell transcription factor 1* (*Utf1)* in SF-gPSCs was similar to that in ESCs and gPSCs and *Oct4*, *Nanog*, *Fgf4*, *Cripto* was higher than in SSCs (Figure 3(A)).

### DNA methylation patterns in SF-gPSCs after expansion on feeder cells

Using bisulfite sequencing analysis, we assessed whether the DNA methylation patterns of SF-gPSCs were altered after reprogramming from SSCs. The promoter regions of *Oct4* and *Nanog* were not methylated in SF-gPSCs, similar to ESCs and gPSCs (Figure 3(B)). The DNA methylation status of multiple CpG sites in the maternally imprinted genes *Small nuclear ribonucleoprotein polypeptide* (*Snrpn*) and *Insulin-like growth factor 2 receptor* (*Igf2r*) was maintained after reprogramming from SSCs into SF-gPSCs (Figure 3(B)).

### *In vitro* and *in vivo* differentiation ability of SF-gPSCs

**Figure 4.**
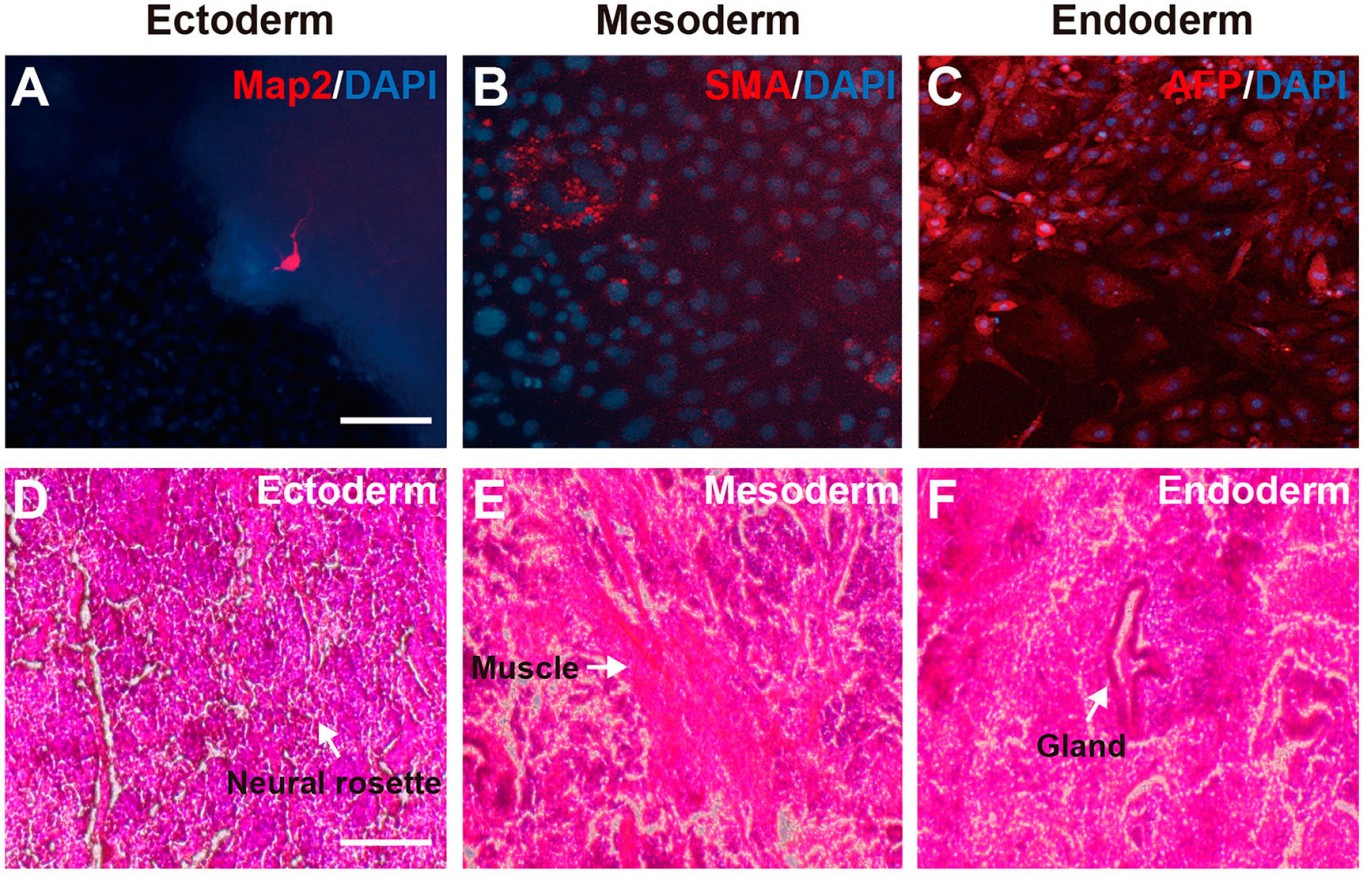
*In vitro* and *in vivo* differentiation of SF-gPSCs. (A–C) *In vitro* differentiation. Immunofluorescence images of cells positive for (A) Map2. (B) SMA. (C) AFP. (D–F) *In vivo* differentiation. Representative images of (D) ectodermal differentiation, (E) mesodermal induction, and (F) endodermal induction. Scale bars: 25 μm (A–C) and 100 μm (D–F).

*In vitro* and *in vivo* differentiation was carried out to confirm the pluripotency of SF-gPSCs. In the *in vitro* analysis, we investigated SF-gPSC differentiation from embryoid bodies into three embryonic layers. After differentiation, we observed positive stained cells for the neuronal cell marker Map2 (Figure 4(A)), the mesenchymal cell marker SMA (Figure 4(B)), and the endoderm lineage marker AFP (Figure 4(C)). To confirm the *in vivo* differentiation potential of SF-gPSCs, they were transplanted into immunodeficient mice to generate teratomas. The presence of teratomas containing three germ layers showed that SF-gPSCs could differentiate into the ectoderm (neural rossette), mesoderm (muscle) and endoderm (gland) (Figure 4(D–F)).

## Discussion

Environmental conditions are important for stem cell culture. Depending on the cell types, various supporting materials such as feeder cells or bio-matrix are required. SSCs have generally been grown on feeder cells. However, recent studies established various feeder-free SSC culture methods using laminin and Matrigel (Kanatsu-Shinohara et al. 2011; Choi et al. 2014). Nevertheless, there is not much research on SSC reprogramming in a feeder-free system. Therefore, we have established the reprogramming of SSCs into pluripotent cells in Matrigel-coated dishes (Lee et al. 2018). However, Matrigel is derived from animal cell extracts and contains a variety of substances. To investigate the mechanism of SSC reprogramming, it was necessary to minimize the presence of exogenous factors. In this study, we prepared 3D scaffolds instead of using coating Material extracted from other organisms. From 3 experiments with 1 × 10^6^ cells per 3D scaffold, we obtained three Oct4-GFP-positive colonies from which two gPSC lines were established. SF-gPSCs showed the same characteristic as ESCs or other gPSCs. Our results confirmed that SSCs can be reprogrammed into pluripotent cells even in 3D spheroids, although the reprogramming rates was lower than for gPSCs in 2D culture. We have also proven that pluripotency can be induced in SSCs without biological materials such as feeder cells or bio-matrix.

It appears that the efficiency of 3D reprogramming of SSCs is lower than that of 2D reprogramming methods, which use either MEFs or Matrigel. We speculate that the reason is due to the fact that unknown exogenous factors existed in MEFs or Matrigel, which may enhance reprogramming of SSCs, are absent in 3D scaffold. Despite of the relatively low efficiency, 3D reprogramming technology can be applied under various conditions, which cannot be done in 2D reprogramming methods. In addition, to study the mechanism of SSC reprogramming in a xeno-free state (i.e. complete absence of exogenous influence), it is essential to use 3D scaffold techniques. In this study, we succeeded in SSC reprogramming under completely xeno-free condition.

## Supplementary Material

Supplemental Table 1

Supplemental Table 2
