# Supplementary material for: Reprogramming of spermatogonial stem cells into pluripotent stem cells in the spheroidal state
Source: Anim Cells Syst (Seoul). 2019 Oct 8;23(6):392–8. doi: 10.1080/19768354.2019.1672578 (PMC6913676; doi:10.1080/19768354.2019.1672578)
Supplement: Supplemental Table 1 [file TACS_A_1672578_SM4147.docx]

**Supplementary Table 1.** Primers for RT-PCR

| Gene | Sequences (5′-3′) |  |
| --- | --- | --- |
| *Oct4* | F-CTG AGG GCC AGG CAG GAG CAC GAG |  |
|  | R-CTG TAG GGA GGG CTT CGG GCA CTT |  |
| *Nanog* | F-AGG GTC TGC TAC TGA GAT GCT CTG |  |
|  | R-CAA CCA CTG GTT TTT CTG CCA CCG |  |
| *Zfp57* | F-ATC ACT TGT GCT GCC AAA GAC |  |
|  | R-CTT CTC CTC CTG GAT TCC ATC |  |
| *Rex1* | F-CAC CAT CCG GGA TGA AAG TGA GAT |  |
|  | R-ACC AGA AAA TGT CGC TTT AGT TTC |  |
| *Utf1* | F-CTC AAG GAC AAA CTC CGA GAC T |  |
|  | R-AGA CTT CGT CGT GGA AGA ACT G |  |
| *Esg1* | F-ATA AGC TTG ATC TCG TCT TCC |  |
|  | R-CTT GCT AGG ATG TAA CAA AGC |  |
| *Fgf4* | F-CAG CGA GGC GTG GTG AGC ATC TTC GGA |  |
|  | R-CTT CTT GGT CCG CCC GTT CTT ACT GAG |  |
| *Eras* | F-TCT GCG TGA CCA GTG CTT GGC |  |
|  | R-TCT TCA GGC TAC AGA GCA GCC |  |
| *Cripto* | F-ATG GAC GCA ACT GTG AAC ATG ATG TTC GCA |  |
|  | R-CTT TGA GGT CCT GGT CCA TCA CGT GAC CAT |  |
| *β-actin* | F-CGT GCG TGA CAT CAA AGA GAA GC |  |
|  | R-ATC TGC TGG AAG GTG GAC AGT GAG |  |

F, forward primer; R, reverse primer
