# Supplementary material for: Reprogramming of spermatogonial stem cells into pluripotent stem cells in the spheroidal state
Source: Anim Cells Syst (Seoul). 2019 Oct 8;23(6):392–8. doi: 10.1080/19768354.2019.1672578 (PMC6913676; doi:10.1080/19768354.2019.1672578)
Supplement: Supplemental Table 2 [file TACS_A_1672578_SM4102.docx]

**Supplementary Table 2.** Primers for bisulfite sequencing

| Gene | Sequences (5′-3′) |  |
| --- | --- | --- |
| *Oct4* (1^st^) | F- GGG ATT TTT AGA TTG GGT TTA GAA AA |  |
|  | R- CCA CCC TCT AAC CTT AAC CTC TAA C |  |
| *Nanog* (1^st^) | F- TTT GTA GGT GGG ATT AAT TGT GAA |  |
|  | R- AAA AAA TTT TAA ACA ACA ACC AAA AA |  |
| *Igf2r* (1^st^) | F- GTA GAG TTT TTT GAA TTT TTT TGT T |  |
|  | R- TAA ACT ATA ATT CTA ATT ATA CCA AAT TAC |  |
| *Snrpn* (1^st^) | F- TAG GTT GTT TTT TGA GAG AAG |  |
|  | R- AAA AAA ACT AAA ACC CCT ACA |  |
| *Oct4* (2^nd^) | F- TGA GGA GTG GTT TTA GAA ATA ATT G |  |
|  | R- AAT CCT CTC ACC CCT ACC TTA AAT |  |
| *Nanog* (2^nd^) | F- TTT GTA GGT GGG ATT AAT TGT GAA |  |
|  | R- AAA AAA ACA AAA CAC CAA CCA AAT |  |
| *Igf2r* (2^nd^) | F- TGG TAT TTT TAT GTA TAG TTA GGA TAG |  |
|  | R- AAA AAT TCT ATA ATC AAA ACC AAC |  |
| *Snrpn* (2^nd^) | F- TAG AGG GAT AGA GAT TTT TGT ATT G |  |
|  | R- ACT AAA ATC CAC AAACCC AACTAA C |  |

F, forward primer; R, reverse primer
